# Recommendations for acceleration of vaccine development and emergency use filings for COVID-19 leveraging lessons from the novel oral polio vaccine

**DOI:** 10.1038/s41541-021-00325-4

**Published:** 2021-04-22

**Authors:** Natalie Thiel, Casey Selwyn, Georgina Murphy, Shmona Simpson, Ajoy C. Chakrabarti

**Affiliations:** 1 University of Washington, School of Public Health, Seattle, WA USA; 2 Bill & Melinda Gates Foundation, Seattle, WA USA

**Keywords:** Biologics, Infectious diseases, Drug development

## Abstract

A new oral polio vaccine, nOPV2, has become the first vaccine to pursue a WHO Emergency Use Listing. Many lessons were learned as part of the accelerated development plan and submission, which have been categorized under the following sections: regulatory, clinical development, chemistry manufacturing and controls, and post-deployment monitoring. Efforts were made to adapt findings from these studies to COVID-19 vaccine candidates. Specific concepts for accelerating COVID-19 vaccine development across multiple functional domains were also included. The goals of this effort were twofold: (1) to help familiarize vaccine developers with the EUL process; and (2) to provide general guidance for faster development and preparations for launch during the COVID-19 pandemic.

## Introduction

The World Health Organization (WHO) Prequalification (PQ) Units’ Emergency Use Listing (EUL) is a unique WHO-facilitated regulatory pathway that can only be used in a declared public health emergency of international concern (PHEIC) or other public health emergency designated by the WHO Director-General^1^. This emergency scenario allows for a product to be listed based on an earlier package of safety and efficacy data than is the norm.

In both 2019 and 2020, the Bill & Melinda Gates Foundation (BMGF) worked closely with multiple product development partners to support P.T. Bio Farma’s submission to the WHO EUL for the novel oral polio vaccine type 2 (nOPV2) vaccine. The nOPV2 vaccine was developed to better address the evolving risk of type 2-circulating, vaccine-derived poliovirus, a risk that elicited a declaration of a PHEIC by the WHO in 2014^2^. The novel vaccine became the first and, to-date, an only vaccine to be submitted for WHO EUL under the revised procedure. It is widely anticipated that a novel COVID-19 vaccine will be the second vaccine type to utilize the WHO EUL procedure^3^.

The EUL will likely play a critical role in accelerating equitable access to COVID-19 vaccines, enabling manufacturing countries to use an emergency pathway to authorize products^1^. Many governments with nascent regulatory systems, such as those in low- and middle-income countries (LMICs), rely on the routine WHO PQ program and the programmatic utilization recommendations to guide procurement and implementation decisions^4,5^. The EUL procedure can be an effective tool for assuring quality and accelerating access to COVID-19 vaccines. Despite this opportunity, not all vaccine developers may be familiar with navigating the WHO EUL for a successful submission.

In this Perspective, we seek to draw on our lessons learned from the nOPV2 WHO EUL experience and provide recommendations to those interested in accelerating COVID-19 vaccine development with the goal of WHO EUL submission. Specifically, we share our observations on key lessons learned in the areas of regulatory, clinical development, chemistry manufacturing and controls (CMC), and post-deployment monitoring with respect-to a WHO EUL. We hope that these insights will help developers effectively plan for WHO EUL interactions and submissions and, thus, accelerate development and global access to safe, effective, quality COVID-19 vaccines.

## Why EUL?

As with many new medical interventions, it is likely that COVID-19 vaccines will be widely available in high-income countries much earlier than in LMICs^6,7^ This issue will be exacerbated if there are vaccine supply constraints, and vaccine manufacturers prioritize high-income country markets. The WHO EUL procedure could help address this potential imbalance by providing a route through which manufacturers, particularly those aiming to supply low-income country markets, can diversify access to their vaccines in the absence of an alternative stringent regulatory agency (SRA) pathway; or, WHO EUL can streamline the process to accelerate global market access following SRA or National Regulatory Agency (NRA) approval.

We acknowledge that some frontrunner COVID-19 vaccine candidates, if successful, are unlikely to follow the WHO EUL and PQ pathways. They may instead opt for approval from an SRA. However, given the unprecedented scale and speed of the need for COVID-19 vaccines, continued vaccine development will likely be necessary to meet global demand^8^. The need to generate affordable vaccines for large-scale, long-term distribution may also be a key driver for continued vaccine innovation. Several promising candidates are currently being developed by Developing Countries Vaccine Manufacturers (DCVMs) at low cost and, for which, the first registration will not be via an SRA. While some vaccine manufacturers may prefer to prepare submissions directly for WHO PQ^9^, the WHO EUL procedure represents an opportunity to accelerate the availability of vaccines by months or, even, years compared to WHO PQ.

**Fig. 1:**
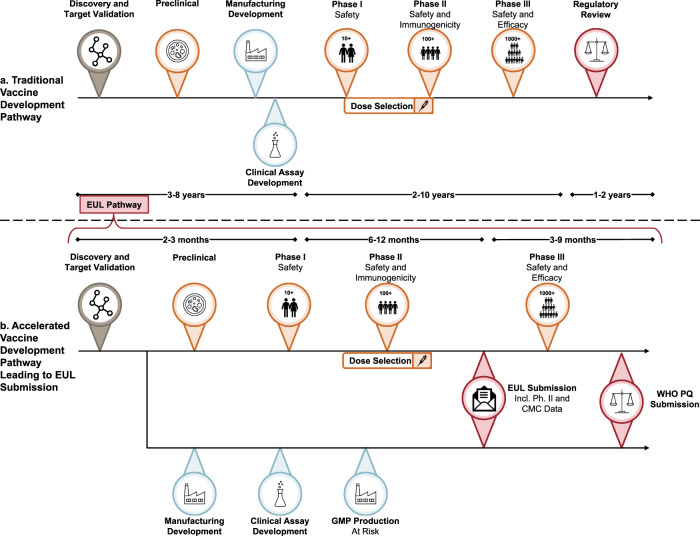
Potential for acceleration across all aspects of development for an EUL submission. Comparison of the traditional drug and vaccine development timeline adapted from Heaton^4^ (**a**) with an idealized vaccine development timeline targeting World Health Organization Emergency Use Listing submission based on nOPV2 and COVID-19 experiences (**b**). Symbols are used to indicate key stages in the vaccine development process; orange-red shading for clinical and blue shading for CMC activities.

Overall, candidates for WHO EUL must demonstrate initial human efficacy (typically phase 2 data), as well as early human safety data. Though a decision is generally made within 90 days, the listing is not intended to circumvent the need for full clinical trial enrollment and analysis, WHO PQ, or other full market authorization processes (Fig. 1b). Under the EUL, a vaccine at an earlier stage of product development might only be listed for use in specific at-risk populations for a time-limited duration, provided the demonstrated benefits continue to outweigh the risks in post-listing studies. Given the high mortality rate and transmissibility of SARS-CoV-2 and the paucity of prophylactic or therapeutic modalities^10–12^, the WHO EUL is clearly suited to the COVID-19 pandemic scenario. Its relatively rapid turnaround time may make the WHO EUL a superior option for accelerated and facilitated regulatory assessment.

In this paper, we describe the recommendations on how to approach a submission for WHO EUL and the accelerated pathways for vaccine development for COVID-19 vaccines based on our observed experience for nOPV2. The WHO continues to provide guidance on their EUL process^3^. It will be important for readers to refer to those resources for the most up-to-date information.

## Regulatory framework

The WHO EUL has pre-emergency, emergency, and post-listing phases^1^. Since the emergency phase begins once a PHEIC is declared, and COVID-19 has already been declared a PHEIC, it follows COVID-19 vaccine development will take place in the emergency phase. While the pre-emergency phase is over, the pre-emergency activities are still critical to successful EUL submission and should be completed during the emergency phase.

### Strategy for the pre-emergency phase

The pre-emergency phase involves establishing platforms for collaborations between the WHO, the subject matter experts, the national regulatory agencies (NRAs) with special expertise, and the NRAs where the products will be used (where they differ). These platforms are leveraged for the pre-submission meetings/activities, product selection, and assessment of submitted data. A key benefit of these platforms is that all regulatory agencies that will be involved in the submission are included as early as possible and, then, throughout the tenure of preparation of the EUL data package. These agencies, WHO, and the manufacturers can align on appropriate non-clinical models and clinical study design, allowing for Phase 2b and Phase 3 trials to commence quickly. This approach can help to define the most relevant clinical trial endpoints for informing EUL by WHO and in target countries^13,14^.

### Strategy for emergency phase

In the emergency phase, it is important for the product developers, regulatory agencies, and subject matter experts to align early on the content and the format of the regulatory submission. Best practices for quickly and efficiently preparing regulatory submissions should be agreed upon. Examples include defining the structure of data tables and developing the document “shells” in advance of clinical data becoming available to enable the rapid completion of data tables and reports. The regulatory strategy should articulate what is the level of authorization (if any) that will be required in the manufacturing country prior to EUL filing. For example, it should be decided whether the product will be submitted to a WHO Maturity Level 4 (ML4) regulator and will, therefore, come to EUL with an emergency authorization from the ML4 regulator^15^. If the vaccine will be manufactured in a country whose regulatory agency is not ML4 but is considered “functional for purposes of vaccine oversight”, it should be determined as early as possible what was the level of authorization (if any) in the manufacturing country that will be required prior to EUL filing. The applicants should clarify which regulatory agency will perform batch release and whether all agencies will rely on that release decision.

The scientific advice and pre-submission meetings are invaluable for reaching an agreement, not only on the content of the submission, but also on the filing strategy. Agreement on the timing and details of a rolling submission, and a review process that allows staggered submission of preclinical, CMC, and clinical data, could be useful in accelerating the overall timeline. If, for example, the CMC and preclinical data are available for review earlier than are the clinical data, a rolling submission could allow assessors to begin and potentially complete their review of those elements sooner than leave it for a later date.

### Strategy for post-listing phase

If the data available at the time of the EUL are considered insufficient to support licensure or WHO PQ, then the developer must reach an agreement with the WHO PQ unit on the nature and timing of data to be submitted for assessment post-listing to support a request for WHO PQ.

A plan to monitor safety and efficacy in the field must be included as part of the EUL submission^16^. However, some countries seeking to adopt the vaccine under EUL may have limited monitoring infrastructure^17^. The NRAs in such countries should engage with experts in safety monitoring and vaccine deployment and with ongoing product assessment and oversight post-EUL under emergency circumstances. Depending on the scientific robustness with which post-listing monitoring data are collected and analyzed, these data may provide primary and/or supplementary data to support decisions on full licensure or removal from the EUL.

### Updates based on emergency status

Following the authorization of the first few COVID-19 vaccines, the ability of later candidates to quality for facilitated regulatory approaches, through the definitions of “unmet clinical need”, will decline: therefore, the rationale for use of the EUL pathway will be impacted. Simultaneously, the applicants will need to demonstrate that COVID-19 remains a PHEIC, perhaps due to supply/demand challenges, or that their product offers superior safety, efficacy, administration, and/or feasibility. Garnering a WHO vaccine program, World Health Assembly resolution, or Strategic Advisory Group of Experts on Immunization recommendation may be important for the procurement agencies and the country’s adoption of the new vaccine under EUL.

## Clinical development

### Overall strategy

When pursuing an EUL, the clinical development group should align with WHO PQ and the primary regulatory authority on the requisite data set in target age groups to support an initial, perhaps restricted, EUL. Specifically, it is crucial to align on the intended wording of the EUL with respect to the target population(s) for use of the vaccine and to include plans for developing data supporting special populations (e.g. children, women of child-bearing age)^18,19^. This will be of particular importance for COVID-19 vaccines which will have a wide-range of target populations and use-cases.

Another strategic goal should be to accelerate clinical studies as much as possible to shorten the timeline to EUL. Table 1 outlines many potential acceleration approaches that can be employed through all stages of clinical development. Several of the strategies address the novel targets and manufacturing approaches associated with COVID-19, which clinical data will help validate.

**Table 1:** Strategies for accelerating clinical development.

Approach	Rationale	Other considerations
Aggressive initial human immunogenicity targets	Multiple candidates may proceed through phase 1 clinical trials. Thus, phase 2 should be designed to enable manufacturers to down-select to one lead candidate. Set immunogenicity endpoints to enable rapid down-selection of competing early-stage candidates and to decrease the likelihood of late-stage immunogenicity failures.	Immunogenicity targets should also determine if a single dose or multiple doses will be necessary, and if single dose vs. two-dose arms should be part of phase 2.
		If a prime-boost is needed, it may be advisable to assess if heterologous prime-boost could provide a more durable response^48^.
Adaptive trial design	Allows for the evaluation of multiple candidates in parallel using pre-determined decision parameters and early efficacy signals to select which arms of the study continue enrollment and which are closed^49^. This enables faster overall clinical assessments and increasing statistical power.	Adaptive trial design has not so far been used for initial vaccine development plans for current COVID-19 vaccine candidates because of the urgency to utilize the available population of clinical subjects but will be advantageous to follow-on vaccine candidates.
Novel biomarkers	Identifying relevant novel biomarkers early in clinical development can accelerate subsequent development.	Examples of useful novel biomarkers have been derived from vaccine studies in animal models of SARS-CoV-2 infection^50,51^
Rapid age escalation and de-escalation	Data in both elderly populations and children will be needed due to the risk of COVID-19 morbidity and mortality in the elderly, and the role of children in spreading the disease^52,53^	Utilize rapid age de-escalation strategies to obtain data in younger populations post-Phase 1^54^.
		Given the lower responses to flu vaccines seen in the elderly, the use of adjuvants, higher dose levels, and/or or novel prime-boost regimens could be helpful to achieve protective immune responses in this population^55^.
		It is important to work closely with regulators to ascertain whether the proposed age escalation to develop data in elderly (50 + years) and more elderly (70 + years) populations needs to be completed prior to EUL; or if the EUL can be amended with new information as the data becomes available.
Considering platform novelty in clinical development	The novelty of the proposed vaccine platform or vector will be key in determining the total number of exposures needed to meet the safety criteria for EUL; and whether regulatory authority alignment will be needed to determine the number of vaccinated subjects needed for the initial human safety database. This needs to be discussed with and agreed with the WHO PQ team and the primary regulator(s).	Novel platforms or vectors will likely require larger exposures/safety databases to allow for widespread use under the EUL, while established platforms may require perhaps only several hundred subjects^56^.
Collecting long-term safety data	Phase 2 subject follow-up assessing response durability and long-term safety is invaluable, and should continue for at least one year after the final vaccine dose is administered.	A proactive plan to update the EUL submission with revised safety and immunogenicity data for long-term follow-up will aid in collecting these data during the EUL submission and review process.
		Follow-up studies should particularly look for any evidence of enhanced disease, and help identify correlates of protection for COVID-19 vaccines^57^.

## Chemistry manufacturing and controls considerations

### Overall strategy

There is no abbreviated CMC EUL path based on risk/benefit criteria^20^. Manufacturers and facilities will be held to established commercial standards, requiring strict adherence to guidelines that are not well-understood by the general public. Large amounts of human and financial capital must be dedicated to programs with high risk of delays or failure.

The CMC strategy should aim for the fastest path to EUL with a plan to manufacture at the scale required to address the pandemic. When pursuing accelerated timelines under heavy public scrutiny, it is important to develop a clear CMC strategy and communication plan. Procurers and implementers need to be aligned with the strategy and commit to it early (even before efficacy readouts) to reduce the risk of supply being delayed or insufficient.

The practices described below are intended to help anticipate and mitigate some of this risk. These recommendations are derived from experience with nOPV2 manufacturing and submission, and are supported by findings from Merck’s experience with their Ebola vaccine manufacturing and submission^21^.

### Regulatory alignment for clinical trials, initial doses, and subsequent use

Manufacturing for a public health emergency must take place much faster than the traditional process development and scale-up^22,23^. EUL CMC standards are based on routine GMP standards for both pilot- and commercial-scale plants^1,24^. It is therefore critical to align with WHO PQ and the relevant regulatory bodies on expectations for standards, documentation, and inspection, as well as process- and analytical-comparability assays and any flexibilities that might apply.

**Fig. 2:**
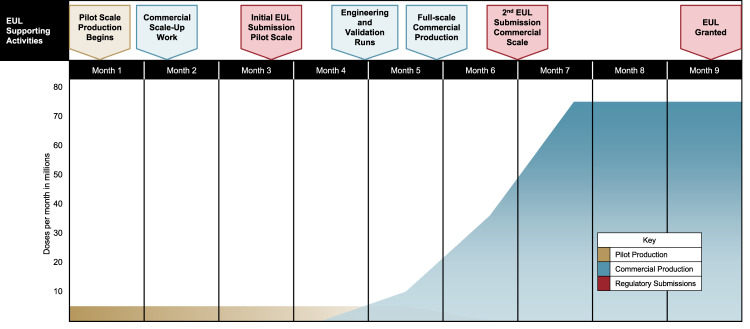
Example of vaccine production for EUL—concurrent activities needed for pilot and commercial facility scale-up and regulatory submissions. Assumptions in this scenario include: rolling EUL submission is allowed, and that pilot-scale process development and engineering and validation runs completed prior to month 1.

Manufacturers and regulators must align on the use of material produced, in pilot-scale and/or alternate facilities, to accelerate the availability of EUL-approved material. Circumstances might require launching doses from pilot-scale and/or alternate-commercial-scale facilities; while commercial-scale and/or additional facilities, commercial processes, and presentations are still being finalized, and, as the regulatory submission is happening (Fig. 2). Because the material used in clinical studies may have been made in a different facility than those being planned for ongoing commercial production, it is necessary to pre-establish parameters for demonstrating adequate process, and analytical comparability, and to continue to improve mechanisms that can accommodate changes while the product is rolled out^25^. In addition, it could be important to plan for a rolling EUL submission that allows for initial review of pilot-scale and/or alternate facilities, with the inclusion of commercial/large-scale production data as it becomes available and validated (Fig. 1).

If possible, manufacturers could agree with WHO PQ and the relevant national authorities on the types of release assays necessary. If these assays require specialized expertise, such as genetic sequencing, it could be beneficial for the manufacturer to bring this capacity in-house early to minimize any testing delays.

### Site selection and capacity planning

Depending on volumes needed, it may be necessary to secure multiple sites for promising candidates, and account for factors such as those outlined in Table 2.

**Table 2:** Factors to consider in site selection and capacity planning.

Factor	Rationale	Recommendations
Containment requirements	The additional regulatory requirements for recombinant viral vector and live viral vaccines can lead to challenges and delays in manufacturing, testing, and distribution^58^.	If possible, regulators could determine whether these classifications will be needed according to appropriate risk/benefit calculations. Developers can then identify facilities that can adhere to relevant standards.
Manufacturer’s expertise	There is wide variety of experience and expertise across manufacturers. Many DCVMs have significant experience navigating the WHO PQ process, the WHO programmatic utilization recommendation process, and GAVI/UNICEF procurement procedures^59,60^. However, several DCVMs have limited experience with novel vaccines. By contrast, many multinational corporations (MNCs) have significant experience developing and manufacturing novel vaccines, but may not be as familiar with WHO PQ, WHO programmatic recommendations, GAVI/UNICEF procurement practices, or with developing packaging appropriate for use in LMICs. Small biotechnology companies are unlikely to have significant experience operating outside of their home country.	For each manufacturer, it is important to plan for the right expertise and technical assistance so that appropriate packaging images are developed, the submission is not incomplete or rate-limiting, analytic processes are established, and procurement procedures are initiated and followed at the right time^61,62^. Other possible areas of assistance include developing new analytical methods for characterization and release, identification and classification of impurities, setting clinically justified specifications, and scaling up to supply global markets.
Multiple, differentiated Backups	Given the high attrition rates inherent in vaccine development, there must be multiple backup plans that optimize for the availability of quality supply upon regulatory approval, programmatic recommendation, and procurement.	Such backup plans require substantial at-risk funding, and the manufacturer will likely need to consider trade-offs in planning and manufacturing for other products, and what level of capacity could be redirected or repurposed if needed^22,25,63^
Inspection planning	Regulatory facility inspections can be challenging to schedule. This issue has been compounded by travel restrictions imposed by COVID-19.	Ideally, a facility could partake in prospective manufacturing inspections for vaccines being developed in response to a PHEIC, where certain parts of the inspection could be conducted virtually, and/or where reliance on a previous PQ or ML4 inspection may suffice under the EUL conditions. For nOPV2, BPOM (Indonesian Food and Drug Authority) assisted with facility inspections typically done by WHO when travel was not possible. The best approach should be discussed early with the WHO PQ unit.
Technology transfer	Vaccine technology transfer requires extensive time and resources^64^. The process includes extensive documentation, asset transfers and training, as well as significant negotiations around intellectual property, liability, and data ownership.	Tech transfer of any vaccine must be planned for as early as possible in the development process, and procurers and implementers need to plan for volumes based on realistic timelines for product availability.
Competent NRA	Under EUL, the facility where the EUL is located may need to assume responsibility for lot-release testing	If the local NRA is unable or unwilling to do this, an alternative will need to be identified.

### Presentation options

Different manufacturing bottlenecks and supply issues may limit flexibility on initial vaccine presentation. For example, adequate drug-substance production but limited filling capacity could require higher dose-per-vial presentations that might not be ideal for routine immunization but could be suitable in campaign settings^26,27^. Maintaining flexibility for changing presentations based on the evolution of the epidemic requires careful consideration of the features in Table 3.

**Table 3:** Presentation features that may change post-initial EUL.

Feature	Recommendation
Stability	• Initiate stability studies for a multitude of dose-per-vial presentations up-front.
	• Because stability studies necessarily require a certain duration, manufacturers may need to negotiate a process for registering with a limited stability claim that is modified as studies of longer duration are completed^65^.
Preservatives	• Need to make decisions about whether to include studies on preservatives.
	• Since not enough time to assess different preservatives is a limitation, initiate discussions with WHO PQ and relevant regulatory authorities on the use of preservative-free, multi-dose vials.
Setting specifications and labelling requirements	• While countries understandably want to set their own specifications and accommodate multiple languages, heterogeneous requirements can result in delays of product availability at the country level.
	• For EUL-approved vaccines that need to be implemented globally, the idea is to harmonize labelling and packaging requirements^21^.
	• Use of QR codes for package labeling, summary of product characteristics/package insert, and patient information leaflet should be discussed with regulators to see if these could be used under emergency circumstances.
Storage/transit infrastructure requirements	• Due to time constraints in process development and optimization, product presentations may have substantial shortcomings in deliverability (e.g. require ultra-cold chain).
	• Trade-offs need to be understood by procurers and delivery-focused groups to help inform where technical solutions and local infrastructure can enable delivery vs. those features that would constitute a no-go^66^.
	• Decisions on presentation should be taken jointly with procurers and delivery-focused stakeholders to ensure feasibility in the field.
	• Can have a plan and process to incorporate different presentation options later without having to re-submit for extensive regulatory review^21^.
Preliminary data generation	• To raise awareness within the field for implementing a novel vaccine, consider using early doses for field studies to establish the effectiveness and early safety data.
	• CMC needs to work closely with implementers to understand implementation options and to solicit early feedback on any component of the presentation that could be modified to aid roll-out and uptake.

### Scaling up/at-risk funding

To ensure sufficient volumes of vaccine are produced and allocated equitably, manufacturers need to be shown the money through some combination of push-and-pull funding^28^. For COVID-19, much of this funding has been supplied through direct government subsidy and commitments to purchase a significant number of doses, post authorization. Some will also be governed by the ACT Accelerator COVAX facility^29^. Currently, there are no UNICEF processes to procure an unlicensed vaccine^30,31^, yet some form of financing is needed to build a stockpile at-risk that can be used upon emergency/conditional authorization (recognizing that the stockpile will have a shelf-life and need to be replenished regularly, even if unused). This risk could be shared between different stakeholders, and might involve some form of a purchase order or guarantee in the event of technical or regulatory failure and/or funding discrete components of long-lead-time equipment and freezers or storage equipment. It may also be useful to procure and stockpile supplies with long shelf-life in non-pandemic times (e.g., syringes)—these items might be impacted by supply chain breakdowns in the pandemic, but do not require highly specialized storage or risk rapid expiration^32^. In addition, to determine the size of investment and reservation, it is important to have reliable demand projections that are backed by purchase orders^33,34^. The stockpile mechanism itself does not own or store doses, which places an additional onus on suppliers to store and prepare vaccines for distribution in future scenarios.

### Cost of goods sold/Pricing for global access

Vaccine pricing must enable broad access, so investments should aim to secure company commitment to pricing at cost of goods sold (COGS) plus a specific margin (COGS + pricing) for LMICs. For scaled, lower-cost access, manufacturers could consider planning for technology transfer to a DCVM or increasing production scale to decrease COGS in the future^35^. Additionally, funders can require contractual definitions for pricing at COGS + pricing at certain volumes.

## Post-deployment product monitoring

### Overall strategy

Ongoing safety monitoring is a key aspect of deploying any new vaccine^36^. A post-deployment monitoring plan is required by WHO for EUL approval. EUL post-deployment product monitoring must include the use of a reporting system for adverse events following immunization (AEFIs), and the option for using active surveillance to investigate specific safety concerns^37^. These systems must report outcomes in a timely manner to both WHO and relevant NRAs.

Like other aspects of the EUL submission, the post-deployment product monitoring plan should be discussed with the WHO PQ unit during pre-licensing meetings to ensure early alignment with EUL requirements. Since EUL candidates must commit to advancing the product to WHO PQ whenever possible, the post-deployment safety monitoring plan should also align with the collection of the remaining clinical data needed for assembling the full WHO PQ dossier.

Strong product monitoring procedures will be crucial for vaccines rolled out under the EUL pathway prior to WHO PQ, or those having full licensure by an SRA. Many COVID-19 vaccine candidates use novel technologies such as an mRNA-based platform^38^, which will require even closer scrutiny because of the lack of historical safety data from similar vaccines^39^. While most high-income countries are equipped with well-established adverse event reporting systems^40^, many LMICs using the EUL pathway lack strong existing reporting systems, making collecting safety data for a newly deployed vaccine in these geographies difficult^41,42^.

### Special considerations for COVID-19

The roll-out of COVID-19 vaccines will be unprecedented in scale and breadth. Never before has the global community attempted to deploy vaccines across so many countries and target populations so quickly. This deployment will be further complicated by multiple vaccines for COVID-19 likely to co-exist under different forms of licensure and authorization.

This unique scenario creates many considerations on how to evaluate risk benefit for different populations, especially in locations with weak safety surveillance systems^43^. Equitable access considerations should be balanced against safety concerns for special populations (such as pregnant women), and the ability to monitor and report AEFIs in the selection of the initial deployment country and populations.

There are also many scientific complexities presented by the multiple vaccine candidate landscape for COVID-19. It is likely that certain COVID-19 vaccines will be rolled out under emergency use authorization while clinical trials for other vaccines are ongoing or in preparation. This presents a challenge on how to design clinical trials for experimental vaccines. Restricting access of trial participants to the available vaccine may be deemed unethical. Thus, the trial design will need to be adaptive, and may be required to compare the experimental vaccine against authorized vaccines rather than placebo.

Alongside safety monitoring, the effectiveness of COVID-19 vaccines will need to be monitored over time. The duration of immunity will not initially be known for new COVID-19 vaccines. Studies will need to be conducted to monitor duration of immunity for each vaccine, post-deployment. Guidance on boosters and the relative performance of different authorized vaccines may subsequently be required.

### Strategies for active surveillance

The considerations above apply largely to passive surveillance of AEFIs. Ideally, comprehensive post-deployment product monitoring of a COVID-19 vaccine would also involve active surveillance and epidemiological studies of vaccine safety outcomes and long-term efficacy^44^. Since pharmacovigilance capacity varies greatly in LMICs^17^, widespread active surveillance of vaccine outcomes in some areas will be challenging. In such areas, there should be additional provision for training to enable active surveillance. Strategies for active surveillance in areas with limited pharmacovigilance capacity include conducting surveillance via sentinel sites such as government immunization centers^45^, leveraging existing epidemiological networks^41^, and implementing targeted cohort studies using local investigators to examine possible safety signals^46,47^.

### Reporting

It will be important for manufacturers to ensure that they have adequate capacity to bring together safety data reported from all geographies in which their vaccines are deployed, whether higher- or lower-income. Those data should be reported to in-country regulators, the manufacturing country’s NRA, and the WHO to ensure that appropriate actions for further investigation can occur.

## Conclusion

The goal of this work was to highlight strategies for streamlining vaccine development and to encourage COVID-19 vaccine developers to consider mechanisms by which they can achieve accelerated access for their products in LMICs. By virtue of BMGF’s involvement with the P.T. Bio Farma nOPV2 EUL submission, we have gained insights into vaccine development acceleration and WHO EUL submission that can be applied to the race for a COVID-19 vaccine and which, we hope, may accelerate the use of the novel vaccines under development to protect at-risk populations. It is anticipated that the vaccine development and global health community will learn a lot about emergency authorization and listing procedures over the next 12–18 months as vaccine candidates for COVID-19 advance. There are many opportunities to leverage these experiences and strengthen procedures for future epidemic preparedness beyond the current COVID-19 pandemic.

## Data Availability

Data sharing is not applicable to this article as no datasets were generated or analyzed.

## References

[1] The World Health Organization. Emergency Use Listing Procedure, Version 8 (WHO, 2020).

[2] The World Health Organization. WHO statement on the meeting of the International Health Regulations Emergency Committee concerning the international spread of wild poliovirus. https://www.who.int/mediacentre/news/statements/2014/polio-20140505/en/ (2020).

[3] The World Health Organization. Roadmap for evaluation of AstraZeneca AZD1222 Vaccine against Covid-19. https://www.who.int/publications/m/item/roadmap-for-evaluation-of-astrazeneca-azd1222-vaccine-against-covid-19 (2020).

[4] Heaton, P. M. The covid-19 vaccine-development multiverse. *N. Engl. J. Med.*10.1056/NEJMe2025111 (2020). 10.1056/NEJMe2025111 PMC7377255 32663910

[5] Pronker, E. S., Weenen, T. C., Commandeur, H., Claassen, E. H. J. H. M. & Osterhaus, A. D. M. E. Risk in vaccine research and development quantified. *PLoS ONE 8*, e57755 (2013). 10.1371/journal.pone.0057755 PMC3603987 23526951

[6] Ahonkhai, V., Martins, S. F., Portet, A., Lumpkin, M. & Hartman, D. Speeding access to vaccines and medicines in low- and middle-income countries: a case for change and a framework for optimized product market authorization. *PLoS ONE 11*, e0166515 (2016). 10.1371/journal.pone.0166515 PMC5112794 27851831

[7] The World Health Organization. Regional Office for Africa. WHO calls for equitable access to future COVID-19 vaccines in Africa. https://www.afro.who.int/news/who-calls-equitable-access-future-covid-19-vaccines-africa (2020).

[8] Corey L, Mascola JR, Fauci AS, Collins FS (2020). A strategic approach to COVID-19 vaccine R&D. Science.

[9] The World Health Organization. Prequalification. http://www.who.int/topics/prequalification/en/ (2020).

[10] Our World in Data. Mortality Risk of COVID-19 — statistics and research. https://ourworldindata.org/mortality-risk-covid (2020).

[11] Siemieniuk RA (2020). Drug treatments for Covid-19: living systematic review and network meta-analysis. BMJ.

[12] UpToDate. Coronavirus disease 2019 (COVID-19): management in hospitalized adults. https://www.uptodate.com/contents/coronavirus-disease-2019-covid-19-management-in-hospitalized-adults?search=covid%2019%20treatment&source=search_result&selectedTitle=1~150&usage_type=default&display_rank=1#H3855514466 (2020).

[13] World Health Organization. Guidelines on clinical evaluation of vaccines: regulatory expectations. https://www.who.int/biologicals/BS2287_Clinical_guidelines_final_LINE_NOs_20_July_2016.pdf?ua=1 (2016).

[14] Hudgens MG, Gilbert PB, Self SG (2004). Endpoints in vaccine trials. Stat. Methods. Med. Res..

[15] World Health Organization. *List of National Regulatory Authorities (NRAs) Operating at Maturity Level 3 (ML3)1 and Maturity Level 4 (ML4)2 (as Benchmarked Against WHO Global Benchmarking Tool (GBT)3*. http://www.who.int/medicines/areas/regulation/nras_ml3_ml4/en/ (WHO, 2020).

[16] World Health Organization. WHO publishes Emergency Use Listing procedure and roadmap to make new medical products more readily available during health emergencies. http://www.who.int/medicines/news/2020/emergency-use-listing-procedure-and-roadmap-he/en/ (accessed Aug 17, 2020).

[17] Olsson S, Pal SN, Stergachis A, Couper M (2010). Pharmacovigilance Activities in 55 Low- and Middle-Income Countries. Drug Saf..

[18] Heath PT, Doare KL, Khalil A (2020). Inclusion of pregnant women in COVID-19 vaccine development. Lancet Infec. Dis..

[19] FDA Center for Biologics Evaluation and Research. Development and Licensure of Vaccines to Prevent COVID-19: Guidance for Industry. https://www.fda.gov/media/139638/download (2020).

[20] World Health Organization. Vaccine quality. https://www.who.int/immunization_standards/vaccine_quality/en/ (2020).

[21] Wolf J (2020). Applying lessons from the Ebola vaccine experience for SARS-CoV-2 and other epidemic pathogens. npj Vaccines.

[22] Lurie N, Saville M, Hatchett R, Halton J (2020). Developing covid-19 vaccines at pandemic speed. N. Engl. J. Med..

[23] Le TT (2020). The COVID-19 vaccine development landscape. Nat. Rev. Drug Discov..

[24] The World Health Organization. Forty-eighth report of the WHO Expert Committee on specifications for pharmaceutical preparations. WHO technical report series; no. 986, Annex 2, 77–136. https://www.who.int/medicines/areas/quality_safety/quality_assurance/expert_committee/ISBN9789241209861-TRS986.pdf (2014).

[25] The World Health Organization. Weekly Epidemiological Record, 7 August 2020, Vol. 95, pp. 369–380. http://www.who.int/wer/2020/wer9532/en/ (WHO, 2020).

[26] Mofrad MH, Maillart LM, Norman BA, Rajgopal J (2014). Dynamically optimizing the administration of vaccines from multi-dose vials. IIE Trans..

[27] The World Health Organization. WHO Target Product Profiles for COVID-19 Vaccines. https://www.who.int/blueprint/priority-diseases/key-action/WHO_Target_Product_Profiles_for_COVID-19_web.pdf (2020).

[28] The World Health Organization. Accelerating access to medicines in a changing world. http://www.who.int/bulletin/volumes/98/9/19-249664/en/10.2471/BLT.19.249664 (2020). 10.2471/BLT.19.249664 PMC7463195 33012865

[29] GAVI, The Vaccine Alliance. COVAX, the act-accelerator vaccines pillar. https://www.who.int/publications/m/item/covax-the-act-accelerator-vaccines-pillar. 2020.

[30] UNICEF. Procurement policies. https://www.unicef.org/supply/resources/procurement-policies (2020).

[31] Yen C (2015). The development of global vaccine stockpiles. Lancet Infect. Dis..

[32] Cundell T, Guilfoyle D, Kreil TR, Sawant A (2020). Controls to minimize disruption of the pharmaceutical supply chain during the COVID-19 pandemic. PDA J. Pharm. Sci. Technol..

[33] UNICEF. Scaling vaccine procurement. https://www.unicef.org/supply/stories/scaling-vaccine-procurement (2020).

[34] UNICEF Supply Division. Rotavirus Vaccine: supply and demand update. https://www.unicef.org/supply/reports/rotavirus-vaccine-rv-supply-and-demand-update. (2020).

[35] Kulkarni PS (2015). Challenges and opportunities while developing a Group A Meningococcal Conjugate Vaccine within a Product Development Partnership: a manufacturer’s perspective from the Serum Institute of India. Clin. Infect. Dis..

[36] Ellenberg SS, Chen RT (1997). The complicated task of monitoring vaccine safety. Public Health Rep..

[37] World Health Organization, Regional Office for the Western Pacific. Immunization Safety Surveillance: guidelines for immunization programme managers on surveillance of adverse events following immunization. http://iris.wpro.who.int/handle/10665.1/126202015 (2016).

[38] Jackson NAC, Kester KE, Casimiro D, Gurunathan S, DeRosa F (2020). The promise of mRNA vaccines: a biotech and industrial perspective. npj Vaccines.

[39] Kochhar S, Salmon DA (2020). Planning for COVID-19 vaccines safety surveillance. Vaccine.

[40] Shimabukuro TT, Nguyen M, Martin D, DeStefano F (2015). Safety monitoring in the vaccine adverse event reporting system (VAERS). Vaccine.

[41] Pirmohamed M, Atuah KN, Dodoo ANO, Winstanley P (2007). Pharmacovigilance in developing countries. BMJ.

[42] Abiri OT, Johnson WCN (2019). Pharmacovigilance systems in resource-limited settings: an evaluative case study of Sierra Leone. J. Pharm. Policy Pract..

[43] Chandler RE (2020). Optimizing safety surveillance for COVID-19 vaccines. Nat. Rev. Immunol..

[44] The World Health Organization. Pharmacovigilance in pilot use of malaria vaccine. http://www.who.int/vaccine_safety/committee/topics/malaria_vaccine/June_2018/en/ (2020).

[45] Vannice KS (2015). Active surveillance for adverse events after a mass vaccination campaign with a Group A meningococcal conjugate vaccine (PsA-TT) in Mali. Clin. Infect. Dis..

[46] Dodoo ANO (2014). Profile of adverse events in patients receiving treatment for malaria in urban Ghana: a Cohort-Event Monitoring Study. Drug Saf..

[47] The World Health Organization. Global manual on surveillance of adverse events following immunization. http://www.who.int/vaccine_safety/publications/aefi_surveillance/en/. (2014).

[48] Lu S (2009). Heterologous prime-boost vaccination. Curr. Opin. Immunol..

[49] Pallmann P (2018). Adaptive designs in clinical trials: why use them, and how to run and report them. BMC Med..

[50] McKay, P. F. et al. Identification of potential biomarkers of vaccine inflammation in mice. eLife 8, (2019). 10.7554/eLife.46149. 10.7554/eLife.46149 PMC6555592 31084714

[51] Bos R (2020). Ad26 vector-based COVID-19 vaccine encoding a prefusion-stabilized SARS-CoV-2 Spike immunogen induces potent humoral and cellular immune responses. npj Vaccines.

[52] Bialek Stephanie (2020). Severe outcomes among patients with coronavirus disease 2019 (COVID-19) — United States, February 12–March 16, 2020. MMWR Morb. Mortal Wkly. Rep..

[53] Szablewski CM (2020). SARS-CoV-2 Transmission and Infection Among Attendees of an Overnight Camp — Georgia, June 2020. MMWR Morb. Mortal. Wkly. Rep..

[54] Kao CM, Orenstein WA, Anderson EJ (2021). The importance of advancing severe acute respiratory syndrome coronavirus 2 vaccines in children. Clin. Infect. Dis..

[55] Weinberger B (2018). Vaccines for the elderly: current use and future challenges. Immune. Ageing.

[56] Iwasaki A, Yang Y (2020). The potential danger of suboptimal antibody responses in COVID-19. Nat. Rev. Immunol..

[57] Plotkin SA (2010). Correlates of protection induced by vaccination. Clin. Vaccin. Immunol..

[58] Drury G, Jolliffe S, Mukhopadhyay TK (2019). Process mapping of vaccines: understanding the limitations in current response to emerging epidemic threats. Vaccine.

[59] The World Health Organization. WHO Prequalified Vaccines. https://extranet.who.int/pqvdata/ (2020).

[60] Hayman, B. & Pagliusi, S. Emerging vaccine manufacturers are innovating for the next decade. *Vaccine X* **5**, 100066 (2020). 10.1016/j.jvacx.2020.100066 PMC7242873 32462140

[61] Plotkin S, Robinson JM, Cunningham G, Iqbal R, Larsen S (2017). The complexity and cost of vaccine manufacturing – an overview. Vaccine.

[62] The World Health Organization. Assessing the programmatic suitability of vaccines candidates for WHO prequalification. http://www.who.int/immunization_standards/vaccine_quality/ps_pq/en/ (2020).

[63] Reuters. Special Report: Countries, companies risk billions in race for coronavirus vaccine. https://www.reuters.com/article/health-coronavirus-vaccine/special-report-countries-companies-risk-billions-in-race-for-coronavirus-vaccine-idUSL2N2CF0JG (2020).

[64] Luter N (2017). An updated methodology to review developing-country vaccine manufacturer viability. Vaccine.

[65] The World Health Organization. Annex 3 Guidelines on stability evaluation of vaccines. http://connection.ebscohost.com/c/articles/84954384/annex-3-guidelines-stability-evaluation-vaccines (2011).

[66] Friend, M. & Stone, S. Challenging requirements in resource challenged environment on a time challenged schedule: a technical solution to support the cold chain for the VSV-Zebov (Merck) Ebola vaccine in Sierra Leone Guinea. *In Proc. of IEEE Global Humanitarian Technology Conference (GHTC)*, 372–376 (2015). 10.1109/GHTC.2015.7343999.

